# Seroprevalence and Associated Risk Factors of Peste des Petits Ruminants in Small Ruminants in North Shewa, Ethiopia: Implications for Eradication Efforts

**DOI:** 10.1155/vmi/3393476

**Published:** 2026-07-22

**Authors:** Enyiew Alemnew Alamerew, Thomas Cherenet, Demeke Sibhatu, Fasil Aklilu, Saddam Mohammed Ibrahim, Firdawok Ayele, Derib Aydefruhim, Asfaw Bisrat, Shenkute Goshme, Yonas Alemayehu, Meron Moges, Melkamu Tadesse, Getachew Hailu, Mastewal Birhan, Anmaw Shite Abat

**Affiliations:** ^1^ Amhara Agricultural Research Institute, Debre Birhan Agricultural Research Centre, Debre Birhan, Ethiopia, arari.gov.et; ^2^ Department of Veterinary Pathobiology, University of Gondar, Gondar, Ethiopia, uog.edu.et; ^3^ Department of Livestock and Fishery Sector Development Project, Minister of Agriculture, Addis Ababa, Ethiopia; ^4^ Department of Serology, Animal Health Institute, Sebeta, Ethiopia, ahi.org; ^5^ Department of Disease Prevention and Control, Minister of Agriculture, Addis Ababa, Ethiopia

**Keywords:** antibody detection, disease eradication, outbreak surveillance, risk-based vaccination

## Abstract

**Background:**

Peste des petits ruminants (PPR) are a highly contagious viral disease that causes substantial morbidity, mortality, and economic losses in Ethiopian small ruminants. Despite ongoing efforts to eradicate PPR by 2027, 554 outbreaks were reported nationwide between 2018 and 2022. This cross‐sectional study, conducted from January to May 2024, estimated the seroprevalence of PPR virus (PPRV) and identified associated risk factors among nonvaccinated small ruminants in six districts of North Shewa zone using competitive ELISA (C‐ELISA).

**Results:**

Of the 324 serum samples tested, 182 were positive for PPRV antibodies, corresponding to an apparent seroprevalence of 56.17% and a true prevalence of 59.18%. Seroprevalence varied across districts, ranging from 27.78% in Menz‐Mama to 92.59% in Menz‐Gera. In the multivariable mixed‐effects logistic regression model, with peasant association (PA) as a random effect, species and district were significantly associated with seropositivity (*p* < 0.05). Goats had approximately four‐fold higher odds of being seropositive than sheep (OR = 4.34; 95% CI: 1.79–12.85; *p* = 0.002), while animals in Menz‐Gera had markedly higher odds compared to those in Menz‐Mama (OR = 37.77; 95% CI: 8.99–158.63; *p* < 0.001). No significant associations were observed for age, sex, body condition score, management system/intervention, or agroecology (*p* > 0.05). Retrospective analysis indicated that 62 vaccination campaigns were conducted in the zone during the study period, yet 48 PPR outbreaks occurred, resulting in 6415 cases and 951 deaths, corresponding to an overall case fatality rate of 14.82%.

**Conclusions:**

PPR appears to remain endemic in North Shewa, with the observed seroprevalence suggesting continued virus circulation, particularly among goats and in high‐risk districts. Despite repeated vaccination campaigns, outbreaks continued to occur during the study period. Therefore, strengthening targeted, risk‐based vaccination, together with improved surveillance and routine seromonitoring, is recommended to support effective PPR control and eradication efforts.

## 1. Introduction

PPR is a highly contagious viral disease affecting domestic and wild small ruminants [1], caused by PPRV, a member of the genus *Morbillivirus* in the family *Paramyxoviridae* [2]. PPRV comprises a single serotype with four lineages (I–IV) [3, 4] and primarily spreads through respiratory droplets, although ingestion of contaminated feed or water can also transmit the infection [5, 6]. Like other morbilliviruses, PPRV is lymphotropic and induces immunosuppression in infected hosts [7, 8]. Clinically, the disease is characterized by fever, depression, anorexia, ocular and nasal discharge, pneumonia, necrosis and ulceration of the mucous membranes, and severe diarrhea [1].

First identified in the Ivory Coast in 1942 [9], PPR has become endemic in many parts of Asia, the Middle East, Sub‐Saharan Africa, and Europe, including Ethiopia [10–12]. It imposes severe economic losses by reducing small ruminant productivity, with morbidity and mortality reaching up to 100% and 90%, respectively, in severe outbreaks. [13]. Globally, approximately 30 million animals are affected annually across 70 countries [14], resulting in losses of up to 2.1 billion USD [15]. In Ethiopia, PPR causes an average loss of US$375 per flock, translating to more than US$2 per animal [16] and significantly impacting food security and rural livelihoods [17].

To mitigate this, the World Organization for Animal Health (WOAH) and the Food and Agriculture Organization of the United Nations (UN FAO) launched a global initiative in 2015 targeting PPR eradication by 2030 [18], while Ethiopia aims for national eradication by 2027 through an RBVS [19, 20]. Despite these efforts, over 70 countries, including Ethiopia, continue to report PPR [14, 21]. Between 2018 and 2022, Ethiopia recorded approximately 554 outbreaks [21], reflecting persistent virus circulation and suboptimal herd immunity, with antibody levels (65.35%–76.66%) often below the recommended ≥ 80% threshold [21–23]. Reported seroprevalence across Ethiopia varies widely (2.1%–75.5%) [24] depending on the region and production system, with notable rates in Tigray (46.53%) [25], Asossa (75.5%) [24], Bale (12.9%) [26], Afar (60.15%) [27], Northwest Ethiopia (32.5%) [28], Dera and Gerar Jarso Districts of Oromia (10.3%) [29], Borena (32.1%) [21], and Amhara (60.8%) [30].

In the North Shewa zone, Ethiopia, small ruminants are important for the agricultural economy and rural livelihoods. However, recurrent PPR outbreaks continue despite ongoing vaccination campaigns [22, 31–33]. Inadequate vaccination coverage and the persistence of susceptible animal populations may contribute to continued virus circulation [22, 31, 34]. Effective control efforts require strengthened vaccination programs, improved surveillance, postvaccination seromonitoring, and enhanced veterinary services [14, 22, 35–38]. Serological surveys in unvaccinated populations are useful for assessing virus exposure and informing targeted control strategies [39]. However, information on the progress of Ethiopia’s risk‐based vaccination strategy (RBVS) in North Shewa zone remains limited, and studies on PPRV seroprevalence and associated risk factors in unvaccinated small ruminants are lacking. Therefore, this study aimed to estimate PPRV seroprevalence and identify potential risk factors among nonvaccinated small ruminants. In addition, retrospective outbreak and vaccination data were analyzed to assess vaccination efforts toward controlling and ultimately eradicating the disease in North Shewa zone.

## 2. Materials and Methods

### 2.1. Description of Study Area

**FIGURE 1:**
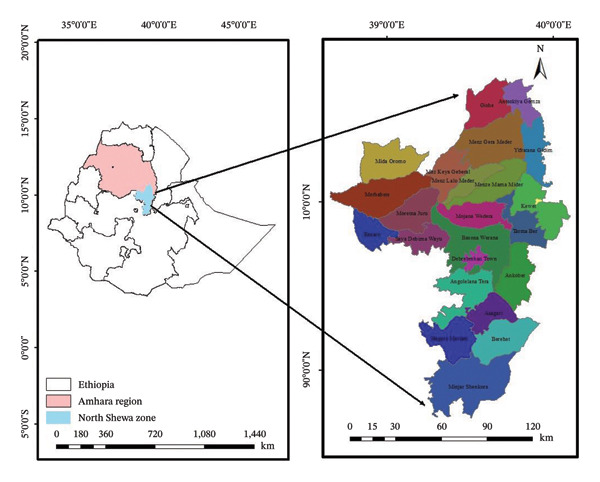
Map of the study areas.

The study was conducted across all 24 districts of the North Shewa zone in the Amhara region, Ethiopia (Figure 1). The zone lies between 9° and 11° N latitude and 38°–40° E longitude, covering approximately 15,936 km^2^ [40]. It borders North Shewa and East Shewa zones of Oromia to the south and southeast, East Gojjam to the west, South Wollo to the north, Oromia Liyu zone to the northeast, and Afar region to the east. Altitude ranges from about 1500 to over 3000 m above sea level, creating diverse agroclimatic conditions. Temperatures range from 10°C to 20°C in the highlands to 20°C–30°C in the lowlands, with annual rainfall ranging from 1085 to 1199 mm. The zone practices mixed crop–livestock farming and hosts approximately 2,738,402 sheep and goats, the second‐largest population in the region, accounting for approximately 3.03% of Ethiopia’s total small ruminant population (90,396,066) [41].

### 2.2. Study Population and Vaccination History

The target population for this study comprised all sheep and goats in the zone, Ethiopia, while the source population included small ruminants accessible within the selected districts and PAs during the study period. Animals were sampled from both intervention and nonintervention areas; however, only animals classified as nonvaccinated were included in the serological analysis to ensure that the results primarily reflected natural virus exposure and existing immunity gaps. Classification of nonvaccinated animals was based on owner‐reported vaccination history, local veterinary records, and the absence of recent vaccination campaigns at both the individual and flock levels, where possible. In intervention areas, sampled animals were those born after the most recent vaccination campaign or those that were below 3 months of age during the last campaign, indicating that they had not been vaccinated. In nonintervention areas, animals were selected from districts where no vaccination campaign had been implemented during the study period (September 2018 to August 2024). To minimize interference from maternal antibodies, only nonvaccinated animals aged 6 months or older were sampled [14]. Animals not originating from the study area, including those obtained through purchase, gifts, or sharing, were excluded. For confirmation of active PPR cases, small ruminants exhibiting clinical signs such as oral lesions, diarrhea, lacrimation, nasal discharge, respiratory distress, and fever (> 40°C) were included.

PPR vaccination in the study area has been implemented under the national RBVS since 2018. This approach targets high‐risk areas based on epidemiological evidence, prioritizing districts with recurrent outbreaks to optimize resource use and reduce virus circulation. Vaccination campaigns are conducted following confirmed outbreaks and primarily focus on at‐risk populations. Under this strategy, suspected outbreaks are investigated by field veterinarians in collaboration with regional coordinators and confirmed using rapid antigen detection tests (pen‐side test kit and dipstick method). Upon confirmation, affected and at‐risk small ruminant populations are isolated and vaccinated using a live attenuated PPR vaccine. At‐risk populations include unvaccinated animals, particularly young stock in previously vaccinated areas or animals with no vaccination history, which are likely to be exposed through animal movement, such as at markets and watering points. Risk classification considers outbreak history, animal movement patterns, population density, and production systems.

### 2.3. Study Design

A cross‐sectional serological study was conducted on small ruminants in six selected districts (Kewet, Shewa‐Robit, Menz‐Gera, Menz‐Mama, Basona‐Werena, and Angolela‐Tera) of the North Shewa zone, Ethiopia, from January to May 2024 to estimate the seroprevalence of PPRV and identify risk factors in unvaccinated animals. A simple random sampling method was used to select the study animals from the population. In addition, a repeated cross‐sectional study was conducted from September 2018 to August 2024 to collect lacrimal and nasal swabs from active cases among small ruminants displaying clinical signs. Furthermore, a retrospective study was undertaken to gather data on the occurrence of PPR outbreaks, case reports, and vaccination campaigns.

### 2.4. Sample Size Determination

The sample size for the seroprevalence study was determined using the following formula [42], assuming an expected disease prevalence of 15.54% [43], a 95% confidence level, and a desired precision of 5%:

(1) n=1.962∗Pex1−Pexd2,

where *n* = required sample size, Pex = expected prevalence (15.54%), *d* = desired absolute precision, and 1.96^2^ = the value of z at a 95% confidence level. Accordingly, the calculated sample size was 202 animals; this was increased by approximately 60%–324 animals (226 sheep and 98 goats) to improve precision and to account for the design effect and potential nonresponse. Animals were selected proportionally from each village based on species distribution.

### 2.5. Sampling Strategy

A multistep selection process was used for the serological study. First, the North Shewa zone was purposively selected due to its implementation of the PPR‐RBVS program [20]. The zone’s 24 districts were then categorized by vaccination status, and four vaccinated districts (Kewet, Shewa‐Robit, Menz‐Mama, and Basona‐Werena) and two unvaccinated districts (Menz‐Gera and Angolela‐Tera) were randomly selected. Two peasant associations (PAs) were randomly chosen from each district, and one village (flock) was randomly selected from each PA, resulting in 12 villages: eight from vaccinated and four from unvaccinated districts. Within each village, animals were selected using simple random sampling, including only unvaccinated animals confirmed by farmers and/or veterinary agents, at least 6 months old, regardless of sex or body condition.

### 2.6. Sample Collection

Approximately 5 mL of blood samples were collected by directly puncturing the jugular vein of selected animals. The blood was allowed to clot; afterward, the serum was separated from the clot and transferred to sterile cryovials, labeled, and transported to the laboratory using a cold chain. The serum was stored at −20°C until testing. During sample collection, relevant information, such as the animal’s origin (district, PA, and village), age, sex, species, and body condition score, was recorded. The recorded body condition of the sheep and goats was grouped into three categories: poor, medium, and good [44]. Also, the age of small ruminants was determined based on dentition into young (six to 12 months), adult (one to 5 years), and old (greater than 5 years) [45, 46]. Although the introduction of new small ruminants from other areas was considered a potential risk factor, at least one animal was added to the study flock by one of these methods. Consequently, it was excluded as a risk factor for PPR seroprevalence.

Furthermore, from September 2018 to August 2024, a total of 192 samples were collected, consisting of 144 lachrymal and 48 nasal swabs. These samples were obtained from 48 outbreak events involving clinically affected small ruminants (sheep and goats). In each outbreak, four samples, three lachrymal and one nasal, were collected from animals exhibiting clinical signs such as oral lesions, diarrhea, lacrimation, nasal discharge (mucopurulent or serous), respiratory distress, and fever exceeding 40°C. Sample collection was carried out using sterile swabs provided in the diagnostic kit. Each swab was gently rubbed against the conjunctiva of the third and lower eyelids, avoiding contact with the eyeball, then placed into a vial containing 30 drops of buffer, and agitated thoroughly to mix.

Retrospective data: in addition to field sampling, retrospective information on PPR outbreaks and vaccination events from September 2018 to August 2024 was collected for the North Shewa zone. This included details on the PPR‐RBVC program, vaccine type, frequency of vaccination event, outbreak, cases, deaths, and populations at risk. A 6‐year dataset on outbreak patterns and vaccination trends was obtained from the North Shewa zone Animal Health Office via the Disease Outbreaks and Vaccination Activity Reports (DOVAR) system. Information on vaccinated and unvaccinated flocks, as well as PA‐level animal lists, was sourced from the zone and district agricultural offices and PA development agents. All data were organized by year and district to reflect outbreak occurrences and vaccination coverage.

### 2.7. Laboratory Examination

#### 2.7.1. Serological Examination

The samples were tested for PPR virus antibodies at the AHI laboratory in Sebeta, Ethiopia, using the ID Screen PPR C‐ELISA kit (IDvet, 310 Rue Louis Pasteur, 34790 Grabels, France), which has a sensitivity of 94.5% and a specificity of 99.4%. This method detects antibodies against the PPR virus nucleoprotein using a validated ELISA kit. Optical density (OD) was measured at 450 nm with an ELISA plate reader, and sample positivity or negativity was determined by calculating the sample‐to‐negative (S/N) ratio according to the manufacturer’s instructions, as follows:

(2) SN=ODSODNC×100,

where ODS is the sample OD and ODNC is the negative control OD, samples with *S*/*N* ≤ 50% were positive, those with 50% < *S*/*N* ≤ 60% were doubtful, and those with *S*/*N* > 60% were negative.

#### 2.7.2. Rapid Antigen Diagnostic Test

Rapid antigen detection was carried out using the Pirbright Institute Peste‐Test UK rapid field test kit, employing two techniques: the pen‐side test kit and the dipstick method. For the pen‐side technique, the device was removed from its packaging, placed on a level surface in the shade, and left for 5 minutes before applying the sample, following the manufacturer’s instructions. For the dipstick technique, either an ocular swab or nasal discharge was used. Twelve drops of buffer were added to the provided tube; the swab was immersed, gently squeezed, and mixed. The dipstick was then inserted in the direction of the arrow and left for up to 20 min. A positive result was indicated by red lines on both the test and control areas.

### 2.8. Data Management and Statistical Analysis

The data from field sampling and retrospective studies were entered into Microsoft Excel for management, data visualization, and calculation of epidemiological parameters, including the frequency of outbreaks, morbidity, mortality, and case fatality rates of PPR. These parameters were calculated using the following formulas: morbidity rate = (number of cases/total population at risk) × 100. Mortality rate = (number of deaths/total population at risk) × 100. Fatality rate = (number of deaths/number of cases) × 100 [42].

Apparent Seroprevalence was determined by dividing the number of animals that tested positive by the total number of animals tested [42]. To estimate the true prevalence, the apparent prevalence (AP) was adjusted based on the sensitivity (Se = 94.5%) and specificity (Sp = 99.4%) of the C‐ELISA test, using the formula described [47]. Adjusted true prevalence values exceeding 100% were truncated to 100% for reporting purposes.

(3) True prevalence=AP+Sp−1Sp+Se−1∗100.

Statistical analyses were conducted using Stata. A null mixed‐effects logistic regression model was initially fitted to assess clustering of PPR seropositivity at the PA level and to determine the necessity of including PA as a random effect in the analysis. The intracluster correlation coefficient (ICC) was estimated using the latent‐variable approach for logistic mixed models as *σ* ^2^ *u*/(*σ* ^2^ *u* + 3.29), where *σ* ^2^ *u* represents the cluster‐level variance, and 3.29 corresponds to the variance of the standard logistic distribution (*π* ^2^/3). In addition, a likelihood‐ratio test was performed to compare the mixed‐effects logistic regression model with the ordinary logistic regression model. Following assessment of clustering effects, univariable mixed‐effects logistic regression analyses were performed to evaluate the association between each explanatory variable and PPR seropositivity, and variables with *p* values < 0.25 were considered for inclusion in the multivariable model. Multicollinearity among explanatory variables was assessed using Spearman’s rank correlation coefficient (*r* ≥ 0.7), and highly correlated variables were excluded based on biological relevance. The multivariable mixed‐effects logistic regression model was then developed using a combination of forward and backward stepwise selection procedures. Two‐way interaction terms were assessed, and confounding was evaluated based on a change greater than 30% in regression coefficients. Statistical significance was declared at *p* < 0.05, and the results are presented as odds ratios (OR) with 95% confidence intervals (CI).

## 3. Results

### 3.1. Seroprevalence

A total of 324 samples were collected across six districts, of which 182 tested positive for PPRV antibodies. This corresponds to an overall AP of 56.17% and a true prevalence of 59.18%, indicating widespread exposure to PPRV among small ruminants in the study area. Among the districts, Menz‐Gera recorded the highest seroprevalence at 92.59%, while Menz‐Mama had the lowest at 27.78% (Table 1). At the PA level, the highest seroprevalence was observed in Sinabanaboda, with 100% positivity, followed by Tsehay‐Sina at 85.19%. In contrast, the lowest was recorded in Zeram PA, with a seroprevalence of 18.52% (Table 1 and 2).

**TABLE 1:** District and PA level seroprevalence for PPRV antibody.

Districts	Peasant associations	No. sampled	No. positive	AP (%)	True prevalence (%)	CI of AP (95%)	
						Lower	Upper
Menz‐Gera	Sinabanaboda	27	27	100	100.00^∗^	87.23	100
	Tsehay‐Sina	27	23	85.19	90.09	66.27	95.81

Basona‐Werena	Abamote	27	12	44.44	46.69	25.48	64.67
	Kormargefiya	27	10	37.04	38.81	19.4	57.63

Kewet	Tere	27	17	62.96	66.41	42.37	80.6
	Yelen	27	20	74.07	78.24	53.72	88.89

Shewa‐Robit	Wustimbay	27	17	62.96	66.41	42.37	80.6
	Wanza	27	15	55.56	58.53	35.33	74.52

Angolelana‐Tera	Bura	27	20	74.07	78.24	53.72	88.89
	Cheki	27	6	22.22	23.02	8.62	42.26

Menz‐Mama	Zeram	27	10	37.04	38.81	19.4	57.63
	Keyafer	27	5	18.52	19.08	6.3	38.08

Total		**324**	**182**	**56.17**	**59.18**	**50.58**	**61.65**

*Note:* The calculated true prevalence exceeded 100% (105.86%) after adjustment for the diagnostic sensitivity (94.5%) and specificity (99.4%) of the C‐ELISA test using the Rogan–Gladen estimator. Since prevalence cannot biologically exceed 100%, the value was reported as 100%.

Abbreviations: AP = apparent prevalence; CI = confidence interval.

^∗^ = 105.86.

**TABLE 2:** Univariable mixed‐effects logistic regression of risk factors for PPRV seropositivity in small ruminants (PA as random effect).

Risk factors	Risk factor category	No. sampled	No. positive	Apparent prevalence (%)	*p* value	Odds ratio	Confidence interval (95%)	
							Lower	Upper
Districts	MENZ‐MAMA	54	15	27.78	Reference	Reference	Reference	Reference
	Basona‐Werena	54	22	40.74	0.272	1.82	0.63	5.29
	Kewet	54	37	68.52	0.001	5.87	1.99	17.44
	Shewa‐Robit	54	32	59.26	0.013	3.90	1.34	11.36
	Angolelana‐Tera	54	26	48.15	0.100	2.46	0.84	7.18
	Menz‐Gera	54	50	92.59	< 0.001	34.74	8.75	137.99

Species	Sheep	226	114	50.44	Reference	Reference	Reference	Reference
	Goat	98	68	69.39	0.002	4.05	1.68	9.80

Age	Young	226	117	51.77	Reference	Reference	Reference	Reference
	Adult	91	67	68.13	0.511	1.27	0.62	2.62
	Old	7	3	42.86	0.723	0.72	0.11	4.52

Sex	Female	227	124	54.63	Reference	Reference	Reference	Reference
	Male	97	58	59.79	0.474	1.23	0.70	2.16

Body condition scores	Poor	43	20	46.51	Reference	Reference	Reference	Reference
	Moderate	160	99	61.88	0.203	1.85	0.76	3.61
	Good	121	63	52.07	0.676	0.84	0.36	1.93

Management	Intervention	216	106	49.07	Reference	Reference	Reference	Reference
	No intervention	108	76	70.37	0.079	3.26	0.87	12.21

Agroecology	Highland	216	113	52.31	Reference	Reference	Reference	Reference
	Lowland	108	69	63.89	0.564	1.51	0.37	6.13

Total		324	182	56.17				

### 3.2. Associated Risk Factors

The null mixed‐effects logistic regression model revealed substantial clustering of PPR seropositivity at the PA level. The estimated between‐cluster variance (*σ* ^2^ *u*) was 1.23, corresponding to an ICC of 27.2%, indicating that a considerable proportion of the variability in PPR seropositivity was attributable to differences between PAs. Furthermore, the likelihood‐ratio test comparing the mixed‐effects logistic regression model with the ordinary logistic regression model was statistically significant (chibar^2^ = 47.02, *p* < 0.001), supporting the inclusion of PA as a random effect in the final model.

The univariable mixed‐effects logistic regression analysis, with PA included as a random effect (Table 2), identified significant associations (*p* < 0.05) between seropositivity and both species and district. Goats (69.39%) had approximately four times higher odds of seropositivity compared to sheep (50.44%) (OR = 4.05; 95% CI: 1.68–9.80). Significant variation was also observed across districts. Compared to Menz‐Mama, animals in Kewet (OR = 5.87; 95% CI: 1.99–17.44; *p* = 0.001), Shewa‐Robit (OR = 3.90; 95% CI: 1.34–11.36; *p* = 0.013), and Menz‐Gera (OR = 34.74; 95% CI: 8.75–137.99; *p* < 0.001) had significantly higher odds of seropositivity. Differences observed in Basona‐Werena and Angolelana‐Tera were not statistically significant (*p* > 0.05). No significant associations were found for age, sex, body condition score, management system/intervention, or agroecology (*p* > 0.05).

Variables with *p* < 0.25 (district, species, body condition score, and management system) were included in the multivariable model. Multicollinearity assessment showed a high correlation between agroecology and species (*r* ≥ 0.7); therefore, species was retained based on biological relevance. No evidence of confounding or significant two‐way interactions was detected. In the multivariable mixed‐effects logistic regression model, with PA retained as a random effect (Table 3), species and district remained significantly associated with seropositivity (*p* < 0.05). Goats had about fourfold higher odds of seropositivity compared to sheep (*p* = 0.002; 95% CI: 1.79–12.85). Similarly, animals in Menz‐Gera had markedly higher odds of seropositivity compared to those in Menz‐Mama (OR = 37.77; *p* < 0.001; 95% CI: 8.99–158.63). Body condition score and management system were not significantly associated with seropositivity (*p* > 0.05), while including PA as a random effect accounted for clustering within sampling units, thereby improving the validity and precision of the estimated associations.

**TABLE 3:** Multivariable mixed‐effects logistic regression model of potential risk factors for PPRV seropositivity in unvaccinated small ruminants (PA as random effect).

Risk factors	Risk factor category	*p* value	Adjusted odds ratio	Confidence interval (95%)	
				Lower	Upper
Districts	Menz‐mama	Reference	Reference	Reference	Reference
	Basona‐Werena	0.289	1.85	0.59	5.78
	Kewet	0.540	1.56	0.37	6.53
	Shewa‐Robit	0.777	1.22	0.31	4.79
	Angolelana‐Tera	0.118	2.49	0.79	7.80
	Menz‐Gera	< 0.001	37.77	8.99	158.63

Species	Sheep	Reference	Reference	Reference	Reference
	Goat	0.002	4.34	1.79	12.85

### 3.3. Retrospective Analysis of Vaccination Efforts and PPR Outbreaks

**FIGURE 2:**
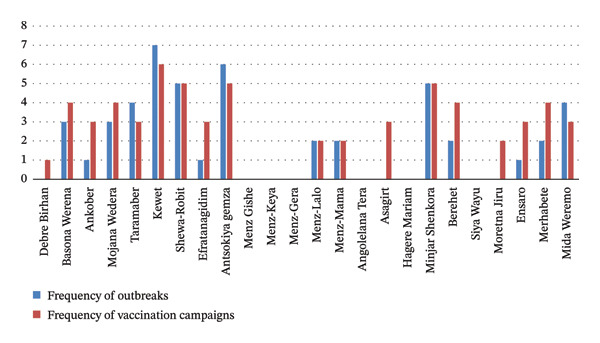
Frequency of PPR outbreaks and vaccination campaigns across study districts during the study periods. Legend numbers of outbreaks (1–7) indicate the number of outbreak events per study district during the study years. Also, legend numbers of vaccination campaigns (1–6) indicate the number of vaccinator visits per district during the study years.

**FIGURE 3:**
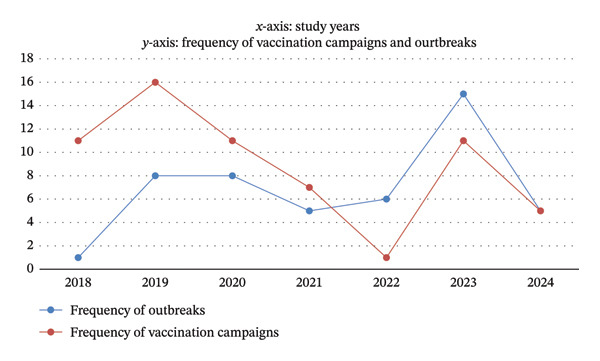
Frequency of PPR outbreaks and vaccination campaigns during the study periods. Legend numbers of outbreaks (1–15) indicate the number of outbreak events per study year across study districts. Also, legend numbers of vaccination campaigns (1–16) indicate the number of vaccinator visits per study year across study districts.

Over the six years, a total of 62 vaccination campaigns were conducted across the study zone. Vaccination frequency varied markedly across districts. Kewet district recorded the highest number of campaigns (six), whereas six districts did not receive any vaccination during the study period (Figure 2). The highest number of vaccination campaigns was observed in 2019 (16 campaigns). In contrast, only one campaign was recorded in 2022 (Figure 3).

Despite ongoing vaccination efforts, PPR outbreaks were reported annually throughout the study period. A total of 48 outbreaks were recorded across 15 of the 24 districts, affecting an estimated at‐risk population of 889,025 small ruminants. Kewet district experienced the highest outbreak frequency (seven outbreaks), while nine districts reported no outbreaks during the study period (Figure 3). Overall, the reported outbreaks resulted in 6415 cases, corresponding to a morbidity rate of 0.72%, a mortality rate of 0.11% (951 deaths), and a case fatality rate of 14.82%. To monitor active outbreaks, 192 clinical samples (144 lachrymal and 48 nasal swabs) were collected from symptomatic sheep and goats, all of which tested positive for PPRV using pen‐side and dipstick diagnostic tests (Figure 2). The temporal distribution of outbreaks showed considerable annual variation. The highest number of outbreaks occurred in 2023, with 15 events. Despite incomplete data for 2018 and 2024, these findings indicate persistent but fluctuating PPR transmission in the study area over time (Figure 3).

## 4. Discussion

### 4.1. Seroprevalence and Epidemiological Significance

Understanding the seroepidemiology and associated risk factors of PPR is essential for effective disease control and eradication strategies. Seroprevalence studies conducted in unvaccinated populations can help identify possible virus circulation and immunity gaps that may not be fully detected through outbreak‐based surveillance alone. Integrating serological findings with retrospective outbreak and vaccination data may provide a broader understanding of progress toward national and global eradication targets under the FAO–WOAH Global Control and Eradication Strategy (GCES) [39]. In the present study, the overall PPRV true seroprevalence was estimated at 59.18%, suggesting widespread exposure to PPRV among small ruminants in the study area. Although this finding may indicate continued circulation of the virus in North Shewa zone, interpretation should be made cautiously, considering the cross‐sectional nature of the study, which does not allow temporal or causal inferences. Nevertheless, the observed seroprevalence highlights the continued epidemiological importance of PPR in the area and its potential implications for small ruminant production and livelihoods.

The current AP (56.17%) is comparable to reports from other regions of Ethiopia, including 55.34% [48], 60.15% in the Afar region [27], and 60.8% in the Amhara region [30], as well as in neighboring Sudan (54.6%) [49]. The consistency of these findings across regions highlights persistent virus circulation in East Africa, likely driven by recurrent outbreaks, incomplete vaccination coverage, and continual replenishment of susceptible animal populations. In contrast, the current AP is higher than findings from other regions of Ethiopia, including 32.5% in Northwest Ethiopia [28], 10.3% in the Oromia region [29], and 32.1% in small ruminants in the Borena zone [21] of Ethiopia. Additionally, the prevalence found in this study is higher than some earlier findings from other countries, such as 42.69% in goat farms in Egypt [50], 27.3% in small ruminants in Uganda [51], 33.48% in small ruminants in Bahrain [52], and 5.3% in goats in Somaliland [12]. On the other hand, the 56.2% seroprevalence reported in this study is lower than some earlier findings, such as 73.45% in Benishangul‐Gumuz [53] and 75.5% in the Asossa zone [24]. Variations among studies may be related to differences in agroecological conditions, animal management practices, vaccination history, animal movement patterns, and study methodologies [54]. Overall, these findings highlight the heterogeneous epidemiology of PPR across different settings and emphasize the importance of context‐specific prevention and control strategies.

### 4.2. Risk Factors Associated With PPRV Seropositivity

The study revealed that animals in nonintervention areas had a higher seroprevalence (70.37%) compared to those in intervention areas (49.07%). Although this difference was not statistically significant (*p* = 0.079, 95% CI: 0.87–12.21), logistic regression suggested a tendency toward higher odds of seropositivity in areas without intervention (OR = 3.26). Specifically, small ruminants aged 6 months or older with no prior vaccination in nonintervention areas were approximately 2.5 times more likely to be seropositive, with a prevalence of 70.4%, compared to 49.1% in vaccinated areas. The higher seroprevalence observed in unvaccinated small ruminants highlights their vulnerability and suggests a potential protective role of vaccination. However, given the cross‐sectional study design and the lack of statistical significance, these findings should be interpreted with caution. These findings reinforce vaccination as the most effective and practical tool for PPR control [55–57], particularly in high‐risk populations, while the observed differences indicate a possible association that warrants further investigation.

In the mixed‐effects logistic regression model, species was a significant risk factor, with goats being 4.34 times more likely to be seropositive (69.4%) compared to sheep (50.4%). This higher susceptibility in goats may reflect species‐specific factors, including differences in immune response and disease exposure. Previous studies have reported that goats are more susceptible to PPRV and tend to experience more severe clinical disease than sheep [27, 28, 48, 58]. Although some studies have reported higher seropositivity in sheep [24, 30, 43, 59–64], such discrepancies may be attributed to differences in breed composition, management practices, and sampling strategies.

The mixed‐effects logistic regression model showed that age was not significantly associated with PPRV seropositivity. Nevertheless, adults (67%) had slightly higher odds of being seropositive, nearly twice as likely, compared to young animals (52.2%). This pattern is likely due to cumulative exposure over time and the prolonged persistence of antibodies following infection [61, 65]. Previous studies in Ethiopia and other endemic countries similarly support the role of age as a proxy for cumulative infection risk [21, 24, 30, 50, 61, 62, 64–71].

The current study identified sex as a nonsignificant risk factor (*p* > 0.05). This sex‐wise antibody prevalence in this study agrees with previous study findings [9, 30, 62, 64, 65] that showed nonsignificance. Even though the difference in the seroprevalence was not significant, previous studies reported higher seroprevalence in female animals [48, 60, 72–74]. The higher seroprevalence in females may be attributed to the practice of retaining females for longer periods for breeding purposes, while males are often sold at an earlier age for income generation. Keeping females for a long time might indirectly account for the detection of high seroprevalence. Stress from pregnancy, kidding, or lambing in females might also contribute to the higher prevalence as a consequence of increased risk of infection.

The prevalence of PPR virus antibodies in the present study showed no significant difference (*p* > 0.05) among small ruminants with varying body conditions. This lack of a significant association is somewhat consistent with previous findings, which suggest that body condition alone may not be a strong indicator of PPR vulnerability [21]. However, this finding contrasts with previous studies [24, 30, 75], which reported higher seroprevalence in sheep and goats with poor body condition. This discrepancy could reflect differences in environmental conditions, management practices, or local disease dynamics.

Marked spatial variation in PPRV seroprevalence was observed across districts and PAs. Small ruminants in Menz‐Gera, a nonintervention district, were nearly 38 times more likely to be seropositive (92.59%) than those in Menz‐Mama (27.78%), where vaccination had been implemented. These findings highlight the strong influence of localized factors such as vaccination coverage, animal density, movement patterns, and management practices on PPR transmission, consistent with earlier studies [28, 49, 76]. Such pronounced heterogeneity underscores the importance of district‐ and community‐level targeting of control interventions [21, 77].

### 4.3. Implications for Eradication Efforts

The PPR GCES uses a four‐stage, stepwise framework to reduce disease risk and guide control efforts. Stage 1–assessment: evaluating the epidemiological situation. Stage 2–control: implementing interventions such as vaccination. Stage 3–eradication: targeting elimination of virus circulation. Stage 4–post‐eradication: confirming the absence of PPR at national or zonal levels to achieve official PPR‐free status from WOAH (OIE). Ethiopia is currently advancing through these stages under the FAO–WOAH GCES framework [14, 36]. According to the PPR Monitoring and Assessment Tool (PMAT) criteria for Stage 1, the findings of this study indicate that North Shewa zone has achieved substantial progress under Stage 1, as evidenced by (i) systematic outbreak reporting, (ii) laboratory confirmation of suspected cases, (iii) retrospective documentation of outbreaks across districts, and (iv) identification of high‐risk areas such as Kewet. Although outbreaks were reported, the relatively low morbidity and mortality rates suggest limited population‐level impact. However, the high seroprevalence among unvaccinated small ruminants (22%–100% at the PA level; 48.2%–92.6% at the district level) indicates ongoing silent virus circulation, suggesting that outbreak‐based surveillance alone underestimates true transmission. These observations highlight the importance of integrating serological evidence into routine monitoring to better inform control strategies, in line with FAO–WOAH recommendations [14, 36].

Progress under Stage 2 is reflected by the implementation of the national RBVS since 2018, through which 62 vaccination campaigns were conducted in the zone [14, 21, 32, 33, 36, 62]. Despite targeted vaccination of high‐risk districts and reduced severity of reported outbreaks, uneven vaccination coverage, rapid population turnover, and animal movement limit the effectiveness of RBVS [31, 78, 79]. Consequently, reported antibody immunity levels in North Shewa (65.4%) remain below the ≥ 80% threshold required to interrupt virus transmission [22], indicating that sustained circulation persists and that transition to Stage 3 has not yet been achieved [14, 36]. Furthermore, the overall AP of 56.17%, together with continued outbreaks and antigen detection, confirms that Stage 4 requirements for verification of freedom from PPR remain unmet, underscoring persistent gaps in proactive vaccination, post‐vaccination seromonitoring, and integrated surveillance systems [22, 31, 80]. Overall, these findings confirm that PPR remains endemic in North Shewa zone, and without systematic integration of serological evidence into vaccination planning and expansion of proactive vaccination to all districts, progress toward elimination and eventual verification of freedom from PPR will remain constrained.

### 4.4. Study Limitations

This study has some limitations that should be considered when interpreting the findings. The cross‐sectional design does not allow for the establishment of temporal relationships or causal inferences between risk factors and PPR seropositivity. Vaccination status classification may be subject to misclassification due to reliance on owner recall and available veterinary records, which could affect the precision of the estimates. Although vaccination records exist for earlier campaigns, more consistent and systematically maintained documentation became available only after the 2018 vaccination campaign implemented under the eradication program. As a result, earlier records may be incomplete, and the possibility of residual or undocumented vaccination, particularly among older animals from nonintervention areas, cannot be fully excluded. Given the long‐lasting immunity induced by the PPR vaccine, this may have contributed to observed seropositivity in some animals. In addition, the use of nonproportional sampling across PAs, without sampling weights due to the absence of population size data, may have introduced some degree of bias in the estimated prevalence. Despite these limitations, strict inclusion criteria were applied, and the findings should be interpreted in light of these considerations.

## 5. Conclusion and Recommendations

This study demonstrated a high seroprevalence of PPRV among nonvaccinated small ruminants in the North Shewa zone, indicating continued circulation of the virus in the area. Species and geographic location were significantly associated with PPRV seropositivity, with higher exposure observed among goats and animals from certain districts. Retrospective data also showed the continued occurrence of PPR outbreaks despite repeated vaccination campaigns conducted during the study period. From an eradication perspective, improvements in outbreak detection and the implementation of risk‐based vaccination strategies appear consistent with progress under GCES Stages 1 and 2. However, the continued circulation of the virus suggests that the level of transmission interruption required for Stage 3 has not yet been achieved. These interpretations are based on descriptive trends observed in the data rather than formal transmission or causal modeling; therefore, conclusions regarding the impact of vaccination on transmission dynamics should be interpreted cautiously. To support ongoing PPR control and eradication efforts, the following recommendations are proposed:

• Improve vaccine distribution and accessibility by implementing a more efficient and equitable system, particularly in remote and underserved areas.
• Refine vaccination strategies by adopting targeted, adaptable schedules that prioritize high‐risk areas.
• Strengthen surveillance systems for continuous monitoring, enabling early detection and timely response to PPR outbreaks, and providing real‐time data to guide vaccination campaigns and control measures.

## Author Contributions

Conceptualization: E.A.A., T.C., D.S., F.A., S.M.I., D.A., F.A., G.H., M.M., Y.A., M.T., M.B., and A.S.A. Methodology: E.A.A., T.C., D.S., F.A., S.M.I., M.B., and A.S.A. Formal analysis: E.A.A. Resources: E.A.A., T.C., D.S., F.A., G.H., M.M., Y.A., M.T., and A.S. Data curation: E.A.A., D.A., and F.A. Writing–original draft preparation: E.A.A. and A.S. Writing–review and editing: E.A.A., T.C., D.S., F.A., S.M.I., F.A., D.A., A.B., S.G., Y.A., M.M., M.T., G.H., M.B., and A.S.A. Supervision: E.A.A., D.S., T.C., F.A., G.H., M.M., Y.A., M.T., and A.S. Project administration: T.C., F.A., G.H., M.M., Y.A., and M.T. Funding acquisition: E.A.A., G.H., and T.C.

## Funding

This research was funded by the Amhara Agricultural Research Institute and the Livestock and Fishery Sector Development Project (LFSDP) of the Ministry of Agriculture, Ethiopia.

## Disclosure

The funders had no specific role in the conceptualization, design, data collection, analysis, decision to publish, or preparation of the manuscript.

## Ethics Statement

The study was conducted with approval from the Institutional Review Board of the University of Gondar under protocol number CVMASc/UoG/RERC/29/12/2023. The experimental protocols complied with the guidelines of the Ethics Committee. This study did not include any experimental treatments or interventions on live animals. Rather, it relied on blood samples that were collected by trained animal health professionals. The sampling process did not cause any additional harm or distress to the animals. Consequently, according to the Animal Health Institute’s guidelines, the study was exempt from formal animal ethical approval.

## Conflicts of Interest

The authors declare no conflicts of interest.

## Data Availability

The data that support the findings of this study are available on request from the corresponding author. The data are not publicly available due to privacy or ethical restrictions.
